# NAMPT-Mediated Salvage Synthesis of NAD^+^ Controls Morphofunctional Changes of Macrophages

**DOI:** 10.1371/journal.pone.0097378

**Published:** 2014-05-13

**Authors:** Gerda Venter, Frank T. J. J. Oerlemans, Marieke Willemse, Mietske Wijers, Jack A. M. Fransen, Bé Wieringa

**Affiliations:** Department of Cell Biology, Nijmegen Centre for Molecular Life Sciences, Radboud University Medical Centre, Nijmegen, The Netherlands; Mayo Clinic, United States of America

## Abstract

Functional morphodynamic behavior of differentiated macrophages is strongly controlled by actin cytoskeleton rearrangements, a process in which also metabolic cofactors ATP and NAD(H) (i.e. NAD^+^ and NADH) and NADP(H) (i.e. NADP^+^ and NADPH) play an essential role. Whereas the link to intracellular ATP availability has been studied extensively, much less is known about the relationship between actin cytoskeleton dynamics and intracellular redox state and NAD^+^-supply. Here, we focus on the role of nicotinamide phosphoribosyltransferase (NAMPT), found in extracellular form as a cytokine and growth factor, and in intracellular form as one of the key enzymes for the production of NAD^+^ in macrophages**.** Inhibition of NAD^+^ salvage synthesis by the NAMPT-specific drug FK866 caused a decrease in cytosolic NAD^+^ levels in RAW 264.7 and Maf-DKO macrophages and led to significant downregulation of the glycolytic flux without directly affecting cell viability, proliferation, ATP production capacity or mitochondrial respiratory activity. Concomitant with these differential metabolic changes, the capacity for phagocytic ingestion of particles and also substrate adhesion of macrophages were altered. Depletion of cytoplasmic NAD^+^ induced cell-morphological changes and impaired early adhesion in phagocytosis of zymosan particles as well as spreading performance. Restoration of NAD^+^ levels by NAD^+^, NMN, or NADP^+^ supplementation reversed the inhibitory effects of FK866. We conclude that direct coupling to local, actin-based, cytoskeletal dynamics is an important aspect of NAD^+^’s cytosolic role in the regulation of morphofunctional characteristics of macrophages.

## Introduction

Important elements of neutrophil and monocyte/macrophage function in innate immunity like cell adhesion, locomotion, phagocytosis and regulation of cell shape are determined by their ability to regulate actin cytoskeleton reorganization [1]. Multiple regulatory principles, established via differentiation programming, play a role in this coupling. After extravasation, monocytes migrate into target tissue areas and differentiate into macrophages. In response to environmental cues, macrophages become polarized, giving rise to different macrophage subtypes. Although other nomenclature has been suggested [2], phenotypically polarized macrophages are broadly classified as classically activated M1 and alternatively activated M2 macrophages [3], [4]. *In vitro*, differentiation toward an inflammatory M1 subtype can be induced by lipopolysaccharide (LPS) stimulation, while IL-4 and IL-13 promote the alternative activation of macrophages towards a suppressive M2 subtype. In keeping with their differential roles in either the initial immune response or the resolution phase of inflammation and tissue healing, the typical morphodynamic activities, including adhesion to the extracellular matrix, or the capturing, adhesion and internalization of cells, debris, or foreign particles via phagocytosis, differ between the M1- and M2-polarized macrophages. Recent work has demonstrated that also the mode of migration, amoeboid or mesenchymal, in 2D and 3D environments differs [5]. However, as pointed out by Cougoule and co-workers it is important to realize that distinction between macrophage shape and motility behavior is often based on *in vitro* studies and that *in vivo*, macrophages may occur in a continuum of phenotypes, with less explicit differences between differentiation-induction effects on morphofunctional properties. Diversity in macrophage phenotype is thus to a large extent the result of variable cues from the tissue microenvironment [6].

One of the most variable environmental cues is nutrient availability which may be dramatically reduced in diseased tissue due to local disruption of the normal blood flow. Macrophages have been shown to sense changes in nutrient availability via sirtuin-1 (SirT1) [7] and modulate the inflammatory response accordingly. Here, we study with a M1 macrophage model whether the metabolic state also has control over one archetypal process in this response, the dynamic remodeling of the actin cytoskeleton that follows induction with bacterial products (like LPS) and the stimulation of phagocytosis by foreign particles [8], [9]. Multiple actin-associated proteins have a role in the spatiotemporal aspects of this remodeling. For proper regulation of actin polymerization and the activity of accessory proteins the cell requires sufficient ATP [10]–[12], supplied via either glycolysis or mitochondrial oxidative phosphorylation (OXPHOS) in a locally demand-controlled manner [13], [14]. That tight regulation of fuel supply is also important for macrophage function is illustrated by the observation that glucose influx through GLUT1 is enhanced when macrophages are exposed to LPS [15], or that glucose consumption via glycolysis is increased when macrophages are incubated with zymosan [16]. Unfortunately, not much more is known about the relative contributions of cytoplasmic and mitochondrial energy-redox reactions to specific morpho-physiological functioning of these cells, although macrophage activities are thought to be generally more dependent on glycolytic metabolism than on oxidative pathways [17]–[19]. In order to produce ATP in sufficient quantities and sustain a high flux through glycolysis, glycolytic cells such as macrophages [20] need to maintain a delicate NAD^+^/NADH balance and threshold concentration of NAD(H) (i.e. oxidized and reduced form) in the cytoplasm. In order to achieve this, NAD^+^ can be synthesized *de novo* from tryptophan, although most of the cellular NAD^+^ in mammalian cells comes from salvage pathways using the NAD^+^ precursors nicotinamide (NAM), nicotinic acid (NA), or nicotinamide riboside (NR) as starting substrates [21]. The first reaction in the conversion of NAM to NAD^+^ is catalyzed by nicotinamide phosphoribosyltransferase (NAMPT) and is the rate limiting step in the pathway, yielding nicotinamide mononucleotide (NMN) as intermediate product.

NAMPT, also known as pre-B cell colony-enhancing factor (PBEF) or visfatin, is one of the more than hundred gene products that undergo conspicuous upregulation upon functional differentiation of macrophages [22], [23]. Apart from having an intracellular enzymatic function in NAD^+^ salvage synthesis, NAMPT is also secreted into the extracellular environment [24]–[26]. Extracellular NAMPT (eNAMPT) appears not to exhibit enzymatic activity but functions as a cytokine by inducing pro-inflammatory responses in macrophages and neutrophils, a role that is unaffected by treatment with the specific inhibitor FK866 (also known as APO866) [27]–[29]. In contrast, inhibition of intracellular NAMPT (iNAMPT) by FK866 decreases intracellular NAD^+^ and LPS-stimulated TNF levels in THP-1 cells and primary mouse and human monocytes as well as IL-1β and IL-6 levels in mouse monocytes [30]–[32]. These observations suggest that a global link exists between NAD^+^ salvage metabolism and the inflammatory response of M1 macrophages. However, whether there is coupling to specific aspects of macrophage functioning or a role of NAD^+^/NADH compartmentalization over mitochondrial and cytosolic pools [33], [34] therein, has not yet been determined.

We have recently, by genetic and pharmacological modulation of NAMPT-dependent NAD^+^ salvage synthesis, provided evidence for a controlling role of NAD(H) (predominantly cytosolic NAD(H)) in the motile behavior of malignant glioma cells [33]. Here we extend this work by extrapolation of these findings to the metabolic control over cellular functions in macrophages. We report on a specific link between cytoplasmic NAD^+^ homeostasis and aspects of adhesion, spreading and phagocytosis in LPS-stimulated cells from the RAW 264.7 lineage and in continuously proliferating MafB/c-Maf deficient (Maf-DKO) macrophages [34]. Pharmacological inhibition of NAMPT was used as a tool to selectively and differentially modulate intracellular NAD^+^ concentration.

## Materials and Methods

### Reagents

FK866 was obtained from Enzo Life Sciences (Antwerpen, Belgium). All other reagents were obtained from Sigma-Aldrich (St. Louis, MO, USA), unless stated otherwise.

### Cell Culture

RAW 264.7 cells (gift from Dr. Hong-Hee Kim, Department of Cell and Developmental Biology, School of Dentistry, Seoul National University, Korea; [35]) were maintained in high-glucose DMEM (Gibco, Life Technologies, Paisley, UK) supplemented with 10% heat inactivated FBS (PAA laboratories, Pasching, Austria), 1 mM sodium pyruvate, and 4 mM GlutaMAX (Gibco, Life Technologies, Paisley, UK), at 37°C in a humidified atmosphere with 7.5% CO_2_. Maf-DKO cells (gift from Dr. Michael H. Sieweke, Centre d’Immunologie de Marseille-Luminy (CIML), Université Aix-Marseille, France; [34]) were maintained in the same way except that medium was supplemented with 20% conditioned medium from L929-cells containing macrophage colony stimulating factor (M-CSF).

### DNA Constructs and Transfection

pEYFP-N1-ΔATG-Lifeact was constructed as follows: Lifeact [36] cDNA, containing human codon sequences flanked by a 5′ BglII and 3′ EcoRI restriction site, was synthesized by GenScript Corporation and provided in a pUC57 plasmid. The Lifeact-fragment did not contain a Kozak sequence, therefore, a forward primer (5′-CT CAG ATC TCC ACC *ATG* GGC GTG GCC GAC C-3′) was designed to induce a BglII site and a Kozak sequence in front of the Lifeact start codon and used together with the M13 universal reverse primer to amplify Lifeact from pUC57 by PCR. PCR products were digested with BglII and EcoRI and ligated into pEYFP-N1-ΔATG plasmid DNA (pEYFP-N1 from Clontech with ATG on position 679 mutated to GCG). For transfection, cells were seeded in 6 well plates at 300 000 cells/well and incubated overnight. DNA (12 µg; linearized with *AseI*) was diluted in 1 ml serum-free DMEM and incubated for 20 minutes at 37°C with 24 µl Targefect-RAW transfection reagent (Targeting Systems, El Cajon, CA, USA). Transfection complexes (250 µl) were added to wells containing 2 ml fresh culture medium and incubated for 4 hours at 37°C after which medium was refreshed. A stable cell population was established by culturing cells for two weeks in medium containing 500 µg/ml G418 and cloning by limited dilution.

### NAMPT and NAPRT Expression

NAMPT and NAPRT expression was analyzed by western blot analysis. Whole cell lysates were prepared using 5x SDS-sample buffer (10% (w/v) SDS, 25% β-mercapto-ethanol, 50% glycerol, 0.05% w/v bromophenolblue, and 312.5 mM Tris-Cl, pH 6.8) and stored at −20°C until analysis. Before lysates were loaded onto 12% SDS-PAGE gels, they were heated at 95°C for 5 minutes. After electrophoretic separation, proteins were blotted onto PVDF membranes. Membranes were blocked with 5% skimmed milk in PBS-T (NAMPT) or TBS-T (NAPRT) for 1 hour and labeled overnight with α-Tubulin (1∶5000; DSHB, University of Iowa) and either α-NAMPT (1∶2500; Bethyl Laboratories, Montgomery, TX, USA) or α-NAPRT (1∶1000; Sigma-Aldrich, St. Louis, MO, USA) antibodies at 4°C. After washing the membranes three times for 5 minutes with PBS-T or TBS-T, they were incubated with IRDye secondary antibodies (GαR800 and GαM680) for one hour at room temperature. This was followed by 4 wash steps of 5 minutes each in PBS-T or TBS-T, a final wash step in PBS, and signal detection on the Odyssey Infrared Imaging System (LI-COR Biosciences, Lincoln, NE, USA). Cell lysate from mouse embryonic fibroblasts (MEFs) was used as positive control for detection of NAPRT.

### NAD(H)/NADP(H) Measurements

Intracellular NAD(H)/NADP(H) was measured according to the protocol published by Wosikowski et al. [37]. Cells (2.0×10^6^) were seeded in 25 cm^2^ tissue culture flasks and incubated in either control medium or medium with 10 nM FK866 for 3, 6, 15, or 24 hours prior to sample collection. Cells were detached mechanically by scraping in fresh culture medium and counted. Suspensions of 2.0×10^6^ cells were spun down and cell pellets were resuspended in 2 ml 0.9% NaCl (Merck, Darmstadt, Germany), split in two and kept on ice. Both fractions were spun down and NaCl was removed. One fraction was used to measure NADH and NADPH levels, and the other fraction was used to measure NAD^+^ and NADP^+^ levels. For NAD(P)H measurement, cell pellets were resuspended in 200 µl 0.02 M NaOH (Merck, Darmstadt, Germany) containing 0.5 mM L-cysteine (Merck, Darmstadt, Germany) and incubated at 60°C for 10 minutes. The alkaline lysates were then neutralized with 60 µl 0.5 M Gly-Gly buffer, pH 7.6, and kept on ice. For NAD(P)^+^ measurement, cells were lysed in 200 µl ice cold 0.5 M perchloric acid (PCA; Fluka) and incubated at 4°C for 15 minutes. The acidic lysates were neutralized by initially adding 80 µl 2 M KOH/0.2 M KH_2_PO_4_, pH 7.5, and adjusting the pH by further addition of 1–5 µl buffer until reaching neutral pH. Extracts were centrifuged at 13,000 rpm at 4°C for 3 minutes. Supernatants were snap frozen in liquid nitrogen and stored at −20°C until analysis. NAD(P)^+^/NAD(P)H was measured spectrophotometrically in an enzymatic cycling assay. The NAD^+^/NADH content of 10 µl sample was measured in 140 µl reaction mix containing 1.8 mM 3-(4,5-dimethylthiazol-2-yl)-2,5-diphenyl-tetrazolium bromide, 70 µM 1-methoxy-5-methyl-phenzzinium methyl sulfate, 64 mM nicotinamide, 0.32 M ethanol, 64 mM Gl-Gly buffer (pH 7.4), 20 mM succinate (SAFC supply solutions), and 20 U alcohol dehydrogenase. The NADP^+^/NADPH content of 10 µl sample was measured in 140 µl reaction mix containing 1.8 mM 3-(4,5-dimethylthiazol-2-yl)-2,5-diphenyl-tetrazolium bromide, 70 µM 1-methoxy-5-methyl-phenzzinium methyl sulfate, 5 mM glucose-6-phosphate, 50 mM Tris-HCl (pH 8.0; Invitrogen), 20 mM succinate, and 0.45 units glucose-6-phosphate dehydrogenase. After addition of reaction mixes, plates were incubated at 37°C for 30–60 minutes and absorbance measured at 510 nm on a BioRad Benchmark Plus micro plate reader. NAD(H) and NADP(H) concentrations were calculated from an NAD^+^ and NADP^+^ standard curve, respectively. Absorbance values were corrected for blank absorbance (without NAD^+^ or NADP^+^) before concentrations were determined. All samples were corrected for the dilution factor.

### NAD(P)H Autofluorescence Measurements

Cellular autofluorescence was determined as a measure of mitochondrial NAD(P)H-level. Cells were seeded in glass-bottom WillCo dishes and incubated with or without 5 nM FK866 for 24 hours. Using the 340 nm filter block, cells were exited for 500 ms and autofluorescence was recorded of at least 10 different fields using the Olympus IX-71 wide field fluorescence microscope (Olympus Mitico) equipped with an EM-CCD camera (Hamamatsu ImagEM). Per field analyzed, the background fluorescence as well as the fluorescence of each cell in the field was measured by determining the average grey value of two regions of interest in the background of the image and of two regions of interest in the cell body. Per cell, the autofluorescence was determined by subtracting the average background fluorescence from the average fluorescence inside the cell. Per condition, 80–110 cells were analyzed.

### Glucose and Lactate Assays

Glucose consumption measurements were based on the Amplex Red Glucose/Glucose Oxidase assay kit from Molecular probes (Life Technologies, Eugene, Oregon, USA). Glucose, glucose oxidase, and Amplex Red reagent were used from the kit but horseradish peroxidase was obtained from Sigma-Aldrich (St. Louis, MO, USA) and 1x reaction buffer was replaced with 0.05 M Tris-HCl, pH 7.5. Apart from these minor changes, the kit protocol was followed as described by the manufacturer. Lactate production was measured using the same protocol as for glucose consumption but replacing glucose oxidase with lactate oxidase and including a lactate standard series instead of glucose. Cells were seeded in 12 well tissue culture plates and incubated for 24 hours in either 1 ml control or 1 ml 5 nM FK866 medium. For glucose measurements, medium containing 5 mM glucose was used while lactate production was measured for cells grown in 25 mM glucose medium. After 18 hours, medium was refreshed in some wells for measurement of glucose consumption or lactate production during the last 6 hours of incubation. Prior to addition of incubation media, wells were always rinsed with pure DMEM containing no glucose. Medium was collected at the end of the 24 hour incubation period and supernatants were snap frozen in liquid nitrogen and stored at −20°C until analysis. Cytosolic extracts were prepared in lysis 100 buffer (50 mM Tris-HCl pH 7.5, 100 mM NaCl, 5 mM MgCl_2_, and 0.5% NP-40; 4°C) and total protein was determined with the Bradford assay. Glucose consumption was calculated by subtracting the amount of glucose in the sample from that in medium without cells. Lactate production was calculated by subtracting the concentration of any lactate in the medium without cells from that of the samples. Glucose and lactate assays were performed in parallel.

### ATP Assay

Intracellular ATP was determined using the CellTiter-Glo cell viability assay kit from Promega. A total of 500,000 cells were seeded per well of a 6-well plate and treated with 5 nM FK866 for 1, 3, or 24 hours prior to assay. Cells were washed twice with ice cold PBS and then scraped in 350 µl ice cold 0.6 M perchloric acid (PCA; Fluka). PCA extracts were centrifuged for 3 minutes at 4000 rpm and 4°C to pellet all cellular protein. Supernatants were neutralized with 140–155 µl 2 M KOH/0.2 M KH_2_PO_4_, pH 7.5 and diluted 1∶10 in water. Per well, 100 µl diluted PCA extract was added to 100 µl CellTiter-Glo reagent and the ATP concentration was determined using an ATP standard series. For determination of total cellular protein, pellets were dissolved in 250 µl 1 M NaOH and heated for 30 minutes at 95°C. Protein concentration was then measured in 1∶50 diluted NaOH extracts.

### Oxygen Consumption Measurements

Mitochondrial respiration was assessed by measuring oxygen consumption on an Oroboros Oxygraph-2 k respirometer according to a standard protocol provided by the manufacturer. Control and 24 hour FK866 treated cells were analyzed in parallel in two separate chambers of the respirometer. After air calibration of control or FK866 medium in the chambers and stabilization of the signal, 1×10^6^ cells (in 60 µl medium) were injected into the respective chambers. Basal respiration rate was measured at the point where the O_2_-flux signal stabilized. Oligomycin (2.5 µM) was added to each chamber and the leak respiration rate was determined after stabilization of the signal. Next, 7 µM carbonyl cyanide-*p*-trifluoromethoxyphenylhydrazone (FCCP, a mitochondrial uncoupler) was added to reach maximal oxygen consumption in the cells. Finally, 30 nM rotenone was added and after stabilization of the system, the residual oxygen consumption could be determined. The data was analyzed using the DatLab software provided with the instrument.

### Proliferation Assay

For cell proliferation measurement, the protocol developed by Skehan et al. [38] was used. Cells were seeded in four 96-well plates (20,000 cells/well) in 100 µl culture medium and incubated overnight. At T0, the medium of plates T6, T15, and T24 were replaced with medium containing 0, 1, 5, or 10 nM FK866 and plate T0 was fixed for sulforhodamine B (SRB) staining of protein content. After 0, 6, 15, and 24 hours of FK866 treatment, cells were washed twice with cold PBS and fixed in 10% trichloroacetic acid (TCA; J.T.Baker, Deventer, Holland) for 1 hour at 4°C. After fixation, plates were washed five times with water and stored at −20°C until all plates were collected. Cellular protein was stained with 50 µl 0.5% SRB in 1% acetic acid for 20 minutes after which wells were washed four times with 1% acetic acid. Plates were dried at 60°C for 3 hours, protein was dissolved in 150 µl 10 mM Tris-HCl (pH 10.5), and the absorbance of each well was measured at 510 nm on a BioRad Benchmark Plus micro plate reader. Values were corrected for background SRB staining by subtracting the average absorbance value of wells that contained medium only, from that of wells with cells.

In order to monitor proliferation up to 72 hours, cells were seeded at a lower density (7,500/well) to avoid any confluency in the cell monolayer and incubated in medium with 10 nM FK866 for 24, 48, and 72 hours. Plates were fixed and stained as described above.

### Apoptosis Assay

Apoptosis of cells was measured using a biosensor (pSIVA) developed by Kim et al. [39]. Briefly, cells (20,000/well) were seeded in a BD Falcon 96-well imaging plate and incubated overnight. On the day of assay, cells were washed once with control or FK866 containing medium before adding 100 µl of fresh control or FK866 medium containing 8 ng/µl pSIVA or non-labeled control (generous gift from Dr. Ralf Langen, University of Southern California). The plate was immediately imaged for 24 hours, continuously, on a BD Pathway high-content spinning disc confocal microscope, using a 20x objective and 2×2 montage capture. Three wells were imaged per condition and the amount of apoptosis was determined by analyzing the increase in GFP-signal. For each well the threshold of the whole GFP-image series was adjusted and the total pixel area/frame was determined using Fiji imaging software and plotted against time.

### Spreading Assay

RAW 264.7 cells expressing Lifeact-EYFP were pre-incubated in control or 5 nM FK866 medium for 0, 3, 15, or 24 hours before they were harvested by treatment with 1 mM EDTA/PBS (10 min at 37°C). After washing, the cells were suspended in medium with 1% BSA. A recovery period of 20 minutes at 37°C was allowed before cells were seeded in a fibronectin coated (50 µg/ml for 2 h at 37°C) BD Falcon 96-well imaging plate at 4000 cells/well. Cell spreading was monitored for three hours by recording the increase in EYFP-pixel area per cell (20 cells/well) on a BD Pathway high-content spinning disc confocal microscope, using a 20x objective and 3×3 montage capture. In order to combine the data from three independent experiments (performed in duplicate), the time axes had to be synchronized. To achieve this, a Boltzmann simulation curve was produced for each data set between time points 0 and 200 in steps of 2 minutes using OriginLab data analysis and graphing software (OriginPro 6.1). Data sets were then combined and analysed for statistical significance by applying a repeated measures analysis using PASW statistics 18 SPSS software. Three time frames (consisting of 30 data points each) of each FK866 curve were compared with the corresponding three time frames of the control curve.

### Scanning Electron Microscopy

Cells were seeded on 12 mm glass coverslips in 24-well plates and treated with 10 nM FK866 for 3, 6, 15, or 24 hours. In addition, cells were activated with 100 ng/ml LPS or left non-activated. Cells were washed once with PBS and fixed with 2% glutarealdehyde in 0.1 M sodium cacodylate buffer for 1 hour. After washing cells twice with cacodylate buffer, coverslips were stored in the same buffer at 4°C until further fixation with 1% OsO_4_ (osmium tetroxide) for 30 minutes. Coverslips were then washed once with water and dehydrated in a graded series of alcohol washes. Finally, coverslips were critical point dried and mounted for scanning electron microscopy (JEOL SEM6340F Field Emission Scanning Electron microscope).

### Cellular Actin Staining

Cells on coverslips were incubated in control or FK866 medium for 24 hours and stimulated with 100 ng/ml LPS overnight or left unstimulated. Cells were washed twice with PBS and fixed in 2% paraformaldehyde in 0.2 M sodium phosphate buffer for 30 minutes. Coverslips were washed twice with PBS and twice with PBS containing 20 mM glycine (MP Biomedicals, Illkirch Cedex, France; PBS-G) before permeabilization with 0.1% saponine/PBS-G for 20 minutes. This was followed by actin staining with Alexa 568-labeled phalloidin (1∶600 in 0.1% saponine/PBS-G) for 1 hour. Cells were successively washed four times for 2–4 minutes with 0.1% saponine/PBS-G and once with PBS alone. Coverslips were removed from wells, rinsed once in water, air dried, and embedded in MoWiol on microscope slides. Z-scans consisting of 25×0.5 µm sections with the pinhole adjusted to 1 airy unit were recorded on a Zeiss LSM510 META confocal laser scanning microscope and merged to one single image using Fiji imaging software.

### Phagocytosis Assay

Phagocytic activity was determined as zymosan ingestion capacity essentially as described by Kuiper et al. [13]. Zymosan particles were dissolved in PBS at 10 mg/ml and left to rehydrate for at least one hour. Next, zymosan was sonicated three times for 5 seconds, spun down, resuspended in carbonate buffer (pH 9.6), sonicated, and incubated with 1 µg/ml fluorescein isothiocyanate (FITC) for 1 hour at room temperature, in the dark. After FITC labeling, zymosan was washed three times with carbonate buffer and incubated in 1 M Tris-HCl, pH 8.0, for 30 minutes. Zymosan was then washed twice with PBS and finally resuspended in PBS. After one more sonication step, FITC-labeled zymosan was divided in aliquots, frozen in liquid nitrogen, and stored at −20°C.

Phagocytosis assays were performed in 12-well plates in which 100,000 cells were seeded per well the day before. Cells were incubated in control or FK866 containing medium for 3, 15, or 24 hours prior to assay and activated overnight with 100 ng/ml LPS. FITC-labeled zymosan particles were complement opsonized by incubation in FBS for 1 hour at 37°C, washed twice with PBS, and finally resuspended in serum-free control or FK866 medium. Cells were washed once with serum-free medium and incubated with 1 ml zymosan suspension for 30 minutes at 37°C. The particle-to-cell ratio was approximately 10∶1. Particle engulfment was terminated by washing cells twice with PBS and removing extracellular zymosan by treatment with 500 µl 100 U/ml lyticase for 10 minutes at room temperature. Successively, cells were detached with 0.05% trypsin/0.5 mM EDTA (Gibco, Life Technologies, Paisley, UK), resuspended in 1 ml medium with serum, pelleted, and finally resuspended in 200 µl 1% paraformaldehyde in PBS. Samples were analyzed by FACS and phagocytosis efficiency was determined by measuring the fluorescence intensity of the FITC positive population as well as the percentage of cells in that population. The phagocytic index of each sample was calculated as the product of the mean FITC intensity of the positive population times the % of FITC positive cells.

To determine the effect of nucleotide supplementation on the phagocytosis efficiency of control and FK866-treated cells, RAW 264.7 cells were incubated for 24 hours with 100 µM NAD^+^, 100 µM NMN or 50 µM NADP^+^ alone or in combination with 5 nM FK866 and phagocytosis efficiency was determined as described above.

### Adhesion and Internalization Assay

Internalization efficiency was determined essentially as described by Sahlin et al. [40]. Cells and zymosan particles were prepared as for the phagocytosis assay. After 30 minute incubation with serum opsonized zymosan at 37°C, plates were transferred to ice and wells were washed twice with ice cold PBS. Cells were then scraped in 1 ml cold medium, divided in two fractions, transferred to microcentrifuge tubes, and spun down. One fraction of each sample was resuspended in 0.05% trypan blue in 13 mM potassium dihydrogen citrate/saline, pH 4.4, to quench the fluorescence of all extracellular particles, and the other fraction in the same buffer but without trypan blue. Samples were analyzed by FACS.

### Statistical Analysis

Data was analyzed either with the Student’s t-test or one-sample t-test for normalized values. All values are expressed as mean +/−SEM. Values were considered to be significantly different when *p* values were <0.05.

## Results

### FK866 Inhibits Cellular NAD^+^ Synthesis

**Figure 1:**
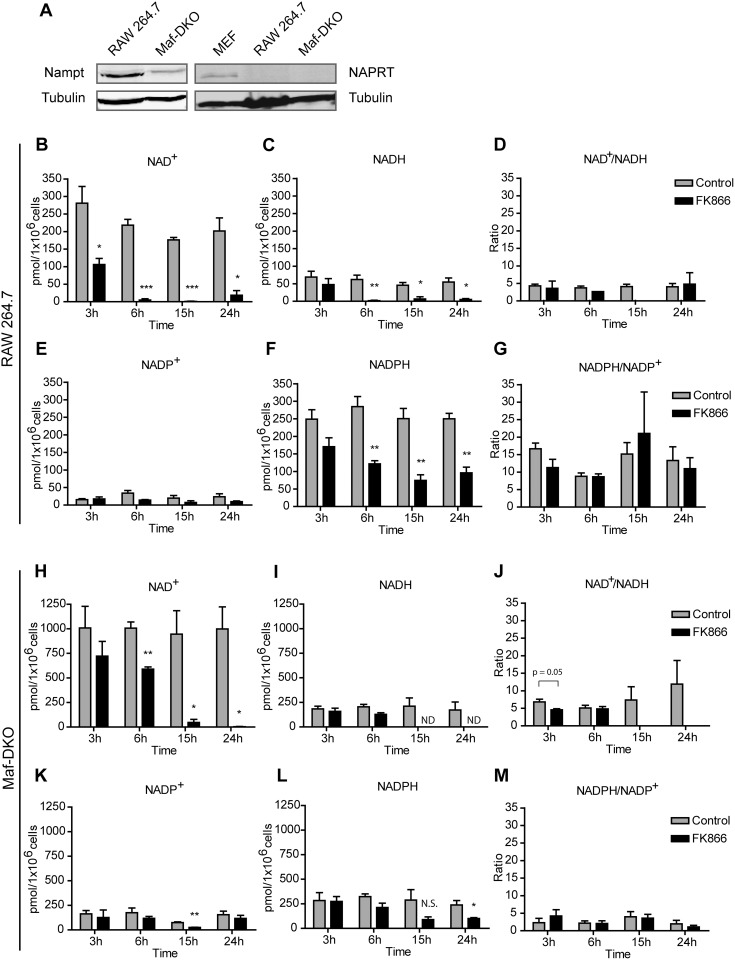
FK866 treatment causes a time dependent decrease in cellular NAD^+^/NADH and NADP^+^/NADPH content. A, Western blot analysis of NAMPT and NAPRT expression in RAW 264.7 and Maf-DKO macrophages. Lysate from mouse embryonic fibroblasts (MEF) was used as positive control for NAPRT expression. Note that relative high amounts of lysate were used (intense tubulin control signal) for detection of NAPRT. B,C,E,F, Pyridine nucleotide levels in RAW 264.7 cells incubated for the indicated time periods with or without 10 nM FK866. H,I,K,L, Pyridine nucleotide levels in Maf-DKO cells incubated for the indicated time periods with or without 5 nM FK866. D,G,J,M, NAD^+^/NADH and NADPH/NADP^+^ ratios were calculated from the data in A&B, E&F, H&I, and K&L. Data in B-M represent means of three experiments performed in triplicate. ND, not detected. (N.S., not significant, *p<0.05, **p<0.01, ***p<0.001, unpaired t-test).

NAMPT mRNA levels in leukocytes are significantly higher than average levels in various mammalian tissues as determined by quantitative real time PCR [24]. However, within the lineage differences exist between leukocyte subtypes in that monocytes and granulocytes express higher NAMPT mRNA and protein levels than lymphocytes. To test if possible variations in mode or capacity of NAD^+^ synthesis could also exist among different macrophage lines, we first determined the relative expression levels of NAMPT and NAPRT, essential enzymes for NAD^+^ synthesis from nicotinamide or nicotinic acid, respectively, in RAW 264.7 macrophages and in *MafB/c-Maf* deficient (Maf-DKO) macrophages [34]. Despite differences in cell origin [34], [41], [42] and proliferative doubling between these macrophage subtypes (Figure S1A), only NAMPT appeared to be expressed at comparable and detectable level (Figure 1A) in both, suggesting that their capacity for NAD^+^ synthesis is similar and primarily determined by the NAMPT reaction step only. Therefore, we considered modulation of NAD^+^ levels by inhibition of NAMPT activity with FK866 a suitable strategy for our further studies into metabolic-morphodynamic relationships in macrophages.

Since NAD^+^ can be converted into other pyridine nucleotides, we compared not only cellular NAD^+^ levels but also determined NADH, NADP^+^, and NADPH levels in the presence and absence of FK866. Strikingly, although most mammalian cells contain at least 5–10 fold more total NAD(H) than NADP(H) [33], [43]–[45], RAW264.7 macrophages and Maf-DKO macrophages appeared relatively rich in NADP(H). In RAW 264.7 cells NAD(H) and NADP(H) levels were about equal (Figure 1B, C, E, F) and in Maf-DKO cells NADP(H) levels were at least one third of that of NAD(H) (Figure 1H, I, K, L). Prolonged FK866 treatment affected NAD(H) and NADP(H) differentially, in a time dependent manner. Within 3 hours, total NAD^+^ levels in RAW 254.7 cells were already reduced by approximately 60% (*p*<0.05; Figure 1B) while NADH, NADP^+^ and NADPH levels remained within the range of the control (Figure 1C, E, F). After 6 hours of FK866 treatment the levels of NAD^+^, NADH, and NADPH were significantly decreased. While NAD^+^ and NADH fell back to levels that were barely detectable, the cellular NADPH concentration remained at 30–40% and did not decline further with prolonged treatment. FK866-mediated NAD(H)-depletion occurred slower in Maf-DKO cells. After 3 hours NAD^+^ levels were not significantly affected and although there was a marked reduction, NAD^+^ was still not fully depleted after 6 hours (Figure 1H). A slower proliferation rate of Maf-DKO cells (Figure S1A), and/or effects of other metabolic differences, may explain the delayed effect of FK866 inhibition in these cells. Nevertheless, upon near complete depletion of cellular NAD(H), after 6 hours for RAW 264.7 and 15 hours for Maf-DKO cells, also NADP(H) levels were profoundly affected. A significant number of cellular reactions depend on the NAD^+^/NADH or NADP^+^/NADPH redox potential, rather than on the absolute concentration of the oxidized and reduced forms of these pyridine nucleotides. We noticed that the NADPH/NADP^+^ ratio fluctuated somewhat (within a two-fold ratio change; Figure 1G, M) during the monitoring period, both with and without the FK866 inhibitor, while this seemed less apparent for the NAD^+^/NADH ratio (Figure 1D, J). However, at some time points (15 h and 24 h) in incubations with inhibitor the NAD^+^ and NADH levels became simply too low to accurately calculate a ratio. Whether the fluctuations observed are an experimental artifact or a feature that is associated with oscillations in cellular redox state, as has been described for yeast cells [46], remains to be determined.

### FK866 Inhibition of NAMPT Reduces Glycolytic Flux but not Intracellular ATP

**Figure 2:**
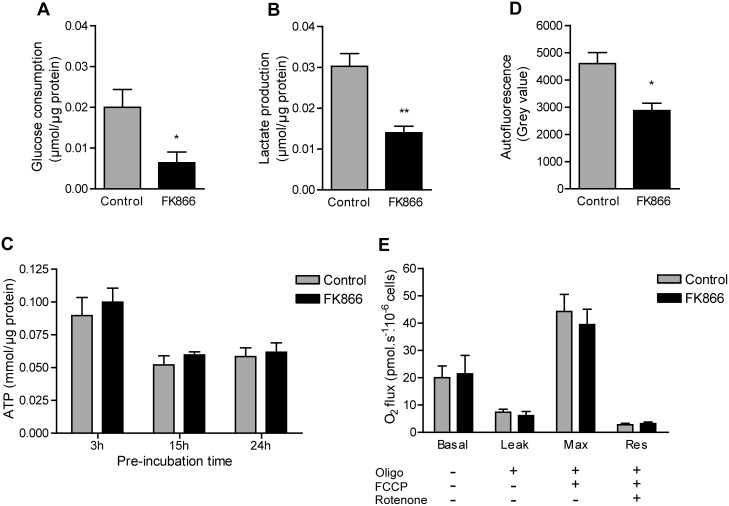
The effect of FK866 on RAW 264.7 metabolism. A, B, Glucose consumption and lactate production in the presence and absence of FK866. Cells were incubated in control or FK866 medium for a total of 24 hours. Glucose and lactate was measured in medium supernatants collected over the last 6 hours (18–24 h) of incubation. C, Intracellular ATP levels of RAW 264.7 macrophages treated with 5 nM FK866 for 3, 15, or 24 hours. D, Mitochondrial NAD(P)H-level. Cellular autofluorescence was measured after 24 hour incubation in control medium or medium with 5 nM FK866. E, After 24 hour pre-incubation with or without FK866, oxygen consumption was measured in suspensions of 1×10^6^ cells in either FK866 or control medium. Control and FK866 treated cells were analyzed in parallel on the same day. The basal oxygen consumption was measured where after oligomycin, FCCP, and rotenone was added successively in order to determine the leak respiration, maximal respiration (Max), and residual oxygen consumption (Res). Data in a-c represent means ± SEM of three experiments performed in triplicate, in d means ± SEM of three experiments, and in e means ± SEM of five experiments. (*p<0.05, **p<0.01; unpaired t-test).

Since the NAD(P)(H) profiles of the two macrophage cell lines were highly similar and FK866 affected the NAD(P)(H) levels to the same extent, we chose to continue our study with main focus on the RAW 264.7 cell line and made comparisons with the Maf-DKO cell line where appropriate. In order to determine the metabolic effect of NAD^+^-depletion we assessed glycolytic activity and ATP production. Other studies already demonstrated that macrophages depend strongly on glycolysis for cellular ATP production [20], [47]. Instead of complete oxidation via the mitochondrial route through the TCA cycle and oxidative phosphorylation (OXPHOS), 80% of the glucose molecules that are imported, are ultimately converted into lactate [19]. For the RAW 264.7 lineage we confirmed this picture and found that ∼75% of all glucose used is converted into lactate, yielding approximately 1.5 moles of lactate per mole glucose (ratio of control bars, Figure 2A, B). In glycolysis, NAD^+^ is converted into NADH by glyceraldehyde-3-phosphate-dehydrogenase (GAPDH), producing 1,3-biphosphoglycerate from glyceraldehyde-3-phosphate. In concert therewith, the end reaction of glycolysis (pyruvate-to-lactic acid conversion by lactate dehydrogenase; LDH), helps to restore the cytoplasmic NAD^+^/NADH balance. Therefore, in view of the crucial role of NAD^+^ availability in maintenance of glycolytic flux, and on the basis of our data shown in Figure 1B, we expected a strong effect of FK866 on glycolytic activity. Metabolic comparison between RAW 264.7 cells cultured in presence and absence of FK866 confirmed this prediction, showing a reduction of 65% and 50% in the amount of glucose consumed and lactate produced, respectively (Figure 2A, B). Simultaneously, the efficacy of lactate formation increased to 1.8 moles of lactate produced per mole of glucose consumed.

In spite of the significant changes in glycolytic activity, we did not observe a significant effect on cellular ATP production capacity. Intracellular [ATP] measured at time points 3, 15, and 24 h after FK866 addition remained in range of the control (around 0.3 µM/µg protein after 3 hours and 0.2 µM/µg protein after 15 and 24 hours; Figure 2C), whereas NAD^+^ levels were already significantly reduced at these time points (Figure 1B). The 30–40% drop in ATP level that occurred in RAW 264.7 cells after 15 and 24 hours was observed repeatedly and can be best explained as a direct result of medium depletion.

### Inhibition of NAMPT-mediated NAD^+^ Synthesis does not Affect Mitochondrial Respiration

A large fraction of the cell’s content of reduced pyridine nucleotides is compartmentalized in the mitochondria. Cellular NAD(P)H autofluorescence is therefore considered a reliable measure for assessing the mitochondrial NAD(P)H status [48]. We utilized this parameter to determine whether FK866 mediated inhibition of NAMPT affected mitochondrial NAD(P)(H) pools of RAW 264.7 cells. Although the autofluorescence intensity of 24 hour FK866-treated cells was reduced by 38% (Figure 2D), it did not mirror the dramatic (60–90%) reduction observed in total cellular NAD(P)H after 24 hours (Figure 1C, F). One possible explanation for this apparent discrepancy may be that FK866 hardly affects mitochondrial NAD(P)(H) levels (see also Pittelli et al. [49]). However, controversy exists around the source of mitochondrial NAD(P)^+^ [50], [51], as the level of this pool appears still to be linked - directly or indirectly - to the level of cytosolic NAD(P)^+^. Therefore, by reducing cytosolic NAD^+^, NAMPT inhibition may ultimately affect the mitochondrial pool, but with differential kinetics.

Another explanation for the roughly one-third shift in autofluorescence signal would be that adaptation in mitochondrial OXPHOS occurs, to compensate for the loss in glycolytic ATP-production. Thereby a situation would be created wherein an increased fraction of total mitochondrial NAD(H) would appear in the (invisible) oxidized form (NAD^+^). In order to obtain a better view on the consequences for mitochondrial respiration capacity, we therefore measured basal oxygen consumption in RAW 264.7 cells pre-treated with 5 nM FK866 for 24 hours and also determined the leak respiration, maximal respiration, and residual oxygen consumption in the presence and absence of FK866. The leak respiration gives an indication of the amount of oxygen that is consumed without producing ATP. The maximal respiration rate is a measure for the respiration capacity, while the residual oxygen consumption after addition of rotenone is a measure for oxygen consumption by systems other than the electron transport chain, for example NADPH oxidases. Figure 2E shows the respiratory values obtained after sequential addition of mitochondrial inhibitors oligomycin, carbonyl cyanide-*p*-trifluoromethoxyphenylhydrazone (FCCP), and rotenone, after measurement of the basal respiration rate. Importantly, we found that FK866 did not affect any of these parameters and therefore left mitochondrial metabolism virtually untouched. We believe, therefore, that the decrease in autofluorescence must represent a reduction in total cellular NAD(P)H, caused by the global FK866-mediated depletion of cytosolic NAD^+^.

### Inhibition of NAD^+^ Salvage Synthesis does not Affect Cell Proliferation or Viability

**Figure 3:**
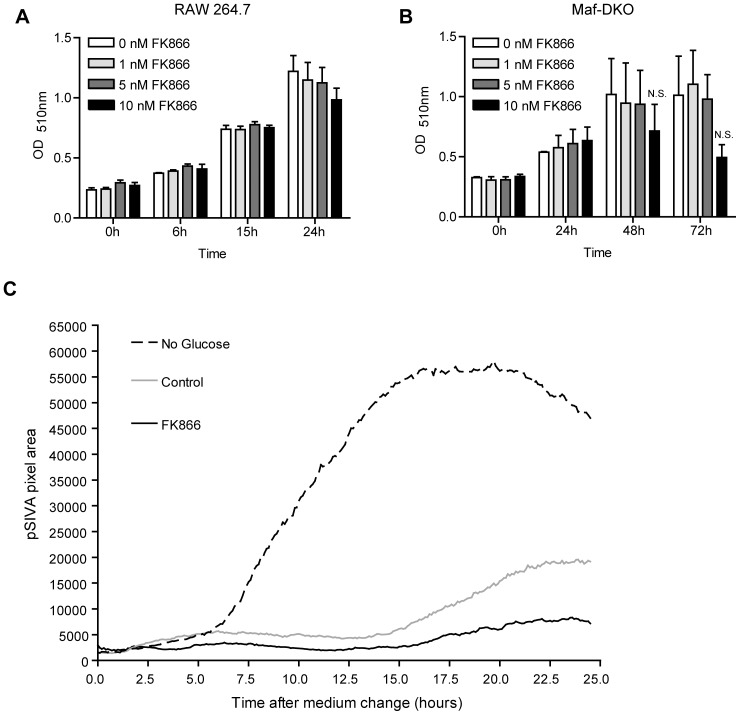
Inhibition of NAD^+^ salvage synthesis with FK866 does not affect proliferation or viability of macrophages. A, B, RAW 264.7 and Maf-KDO proliferation in the presence of different concentrations of FK866, monitored by measuring the increase in protein mass at different intervals over a 24 or 72 hour period. Data represent means of three independent experiments performed in triplicate. C, RAW 264.7 cell viability in the presence of 10 nM FK866. The appearance of the fluorescent apoptosis-specific pSIVA signal was recorded over time and the total pSIVA pixel area was determined per frame using Fiji Imaging software and plotted against time. Lines represent averages from one experiment performed in triplicate. (N.S., not significant; unpaired t-test).

Next, we investigated the effects of blockade in NAD^+^ salvage synthesis on cell viability and proliferation capacity. Figure 3A shows the increase in total protein mass as a consequence of growth in RAW 264.7 cell populations, cultivated in absence or presence of different concentrations of FK866. Within the entire 24 hour period of incubation, FK866 at concentrations up to 10 nM had no - or only a very minor - effect on the rate of proliferation of RAW 264.7 macrophages. Due their slower growth rate, proliferation of Maf-DKO cells was monitored for 72 hours and also found not to be affected at these concentrations up to 48 hours (Figure 3B). Furthermore, use of a polarity-sensitive annexin-based apoptosis biosensor (pSIVA) with switchable fluorescence states [39] also did not reveal an increase in the frequency of RAW 264.7 apoptosis within the 24 hours of FK866 treatment (Figure 3C). In contrast, upon cultivation in glucose-free medium, used as a control-condition for apoptosis induction, pSIVA signal strength increased strongly, indicating that RAW 264.7 cells underwent massive apoptosis within 10 hours. Together, these observations indicate that cell viability and cell growth are not compromised within the test period and under the conditions used for inhibition of NAD^+^ salvage synthesis with FK866.

### LPS-stimulated Spreading and Formation of Actin-rich Membrane Protrusions are Inhibited in FK866 Treated Macrophages

**Figure 4:**
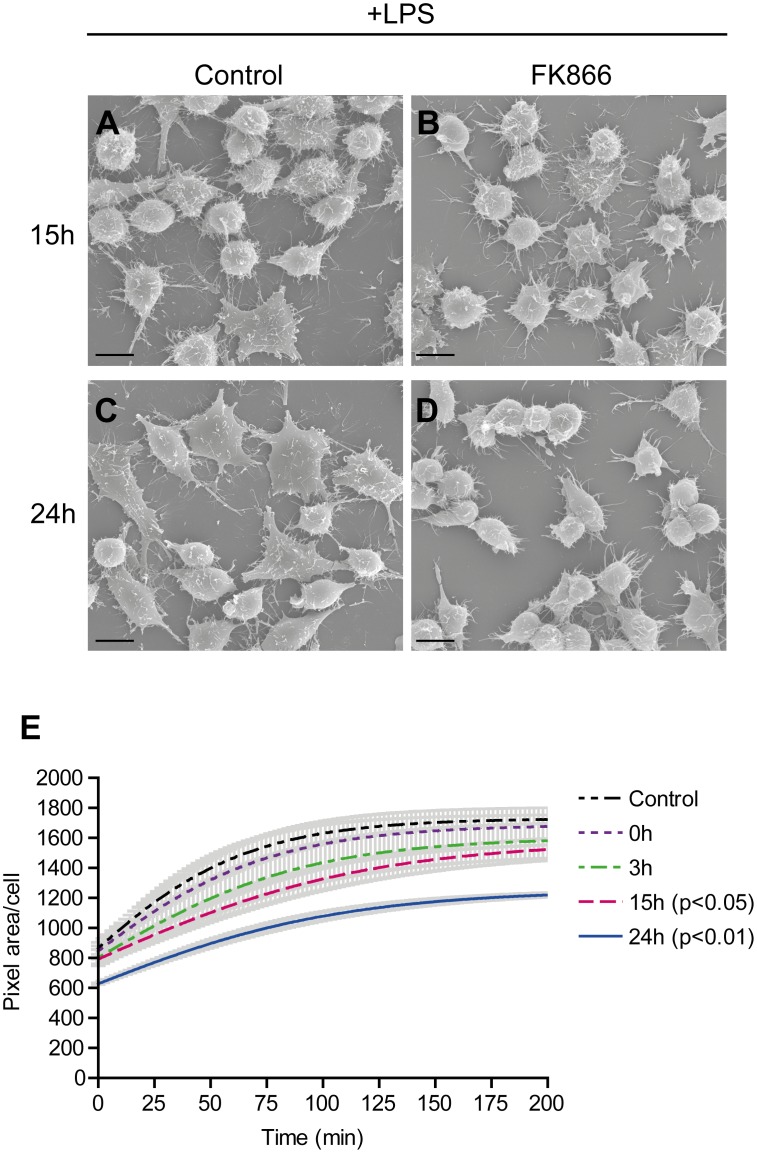
LPS-stimulated spreading is inhibited during NAMPT inhibition in RAW 264.7 macrophages. A–D, RAW 264.7 cells were treated with 10 nM FK866 for 15 or 24 hours and simultaneously stimulated overnight with LPS. A representative image of each condition is shown, (Bar = 10 µm). E, To quantify spreading efficiency, RAW 264.7 macrophages expressing lifeact-EYFP were pre-treated with 5 nM FK866 for the indicated time periods, detached, resuspended, seeded in 96 well plates and allowed to adhere. Adherence and spreading of EYFP-positive cells were recorded over time. The average pixel area per cell was determined at every 10 minute interval. Lines represent means ± SEM (grey bars) of three experiments performed in duplicate.

The immune activity of macrophages requires the dynamic remodeling of their cortical actin cytoskeleton, active transport of membrane components, and a diverse set of receptor-ligand binding reactions at the cell surface. Together, these are processes with a large cellular energy demand [10], [11], [13]. Remodeling of the cytoskeleton and engagement in adhesion to particles, or binding to other cells or the ECM are also under control of other aspects of metabolism, including calcium and redox homeostasis [52]–[56]. In order to disclose possible involvement of NAD^+^ in the morphofunctional behavior of macrophages we first analyzed whether NAMPT inhibition affected cell surface morphology. Scanning electron microscopy (SEM) images of resting RAW 264.7 cells after different FK866 incubation periods revealed no differences compared to control cells (not shown). Next, we analyzed the adhesive ability of cells with normal or impaired NAD^+^ salvage capacity after LPS-stimulation. Interestingly, the ability to undergo spreading and the formation of LPS-induced cellular protrusions appeared affected by NAMPT inhibition. Cells treated with FK866 failed to spread upon LPS stimulation already after 15 hours (Figure 4A, B) but the effect was most prominent after 24 hours (Figure 4C, D). In order to quantify the effect of FK866 on cell spreading, we followed the adhesion and spreading of RAW 264.7 cells expressing Lifeact-EYFP in real time. The cells were stimulated with LPS overnight and pre-incubated with 5 nM FK866 for 0, 3, 15 and 24 hours after which they were seeded in 96 well plates and allowed to adhere. FK866 clearly inhibited the spreading ability of the cells in a time dependent manner (Figure 4E). Three hour pre-incubation with FK866 only had a minor affect, while spreading was significantly impaired after 15 and 24 hours of NAMPT inhibition. A notable observation was that 24 hour FK866-treated cells already covered a smaller cellular pixel area at T0. This may indicate that NAMPT inhibition affected the pliability of the cells, causing them to maintain a rounded shape after reaching the well bottom instead of undergoing ventral flattening. Alternatively, it may indicate that the formation of cellular adhesion structures has been disturbed. Using TIRF microscopy we attempted to detect differences in the formation of actin-based adhesion structures between control and FK866-treated cells but, unfortunately, our results were inconclusive.

**Figure 5:**
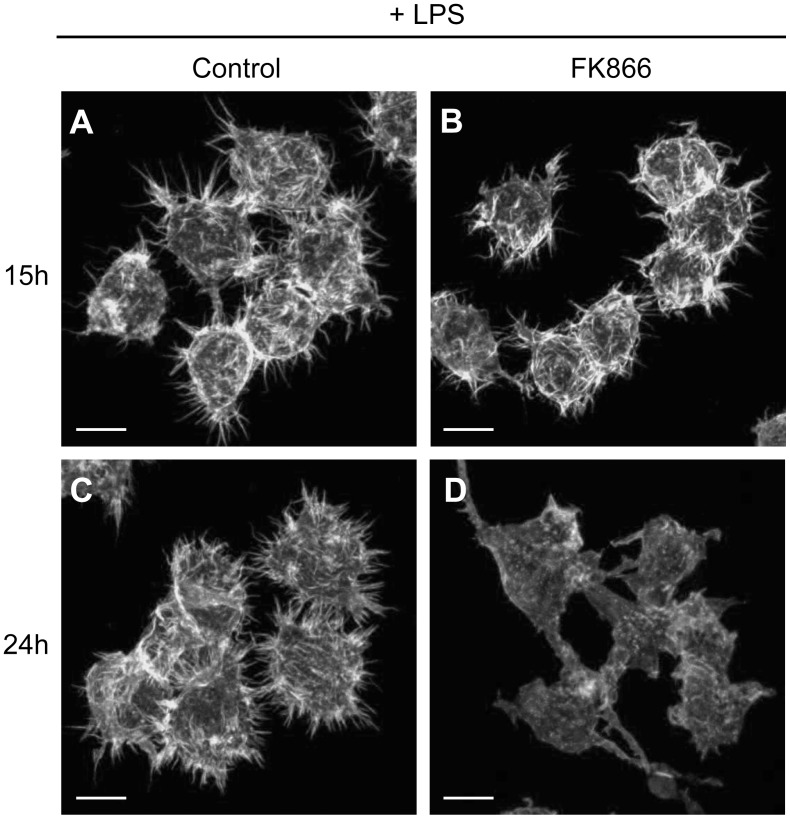
Actin reorganization is altered during NAMPT inhibition in RAW 264.7 macrophages. Actin organization was assessed in wild type cells seeded on glass coverslips, treated with FK866 for 15 or 24 hours, and simultaneously stimulated overnight with 100/ml LPS. After fixation in 2% PFA, cellular actin was stained with phalloidin-Alexa568 and cells were imaged on a Zeiss LSM510 META confocal laser scanning microscope. (Bar = 10 µm).

To know otherwise whether the organization of the actin cytoskeleton of the cells was affected by FK866, cells were again stimulated overnight with LPS and treated with FK866 for 3, 6, 15, and 24 hours. After this treatment they were fixed and stained with Alexa568-labelled phalloidin. No difference was observed in the actin cytoskeleton after 3 and 6 hours of FK866 treatment (not shown). However, after 15 hours and especially after 24 hours, the formation of actin-rich membrane structures was disrupted in FK866 treated cells. Control cells developed multiple cellular protrusions upon LPS stimulation with a dense and straight appearance, while this was less well visible or even absent in the FK866 treated cells (Figure 5A–D). Phalloidin staining of Maf-DKO cells revealed that the cells themselves as well as their actin-rich surface structures have a strikingly different morphology compared to RAW 264.7 cells (Figure S2A, E). Nevertheless, upon LPS-stimulation, Maf-DKO control cells also developed sharp membrane protrusions which were, of note, also inhibited in the presence of FK866 (Figure S2A–H). Blocking of NAD^+^ salvage synthesis therefore clearly affects LPS-induced macrophage morphology, spreading ability, and the formation of actin-rich membrane protrusions.

### Phagocytosis of Serum Opsonized Zymosan is Hampered when NAD^+^ Salvage Synthesis is Inhibited

**Figure 6:**
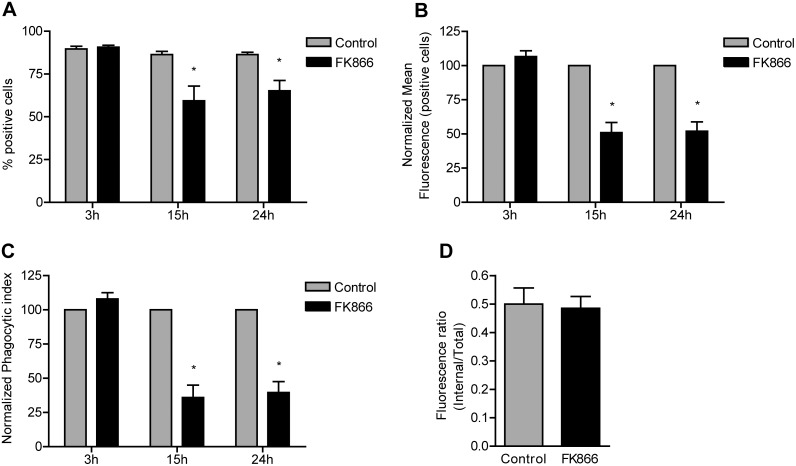
Phagocytosis efficiency is reduced when NAD^+^ salvage synthesis is inhibited. Cells were pretreated with 10-labeled complement opsonized zymosan (COZ) particles for 30 min and analyzed by FACS. For each sample the percentage of FITC positive cells (A) and mean fluorescence of FITC positive cells (B) were measured. The phagocytic index (C) was calculated as the product of these two parameters. The internalization efficiency (D) was determined by quenching the extracellular FITC-COZ of one sample fraction with 0.05% trypan blue in potassium dihydrogen citrate/saline, pH 4.4. Unquenched fractions were used to determine the total fluorescence per cell (internalized and external particles), while quenched fractions were used to measure only the internal fluorescence per cell. Data represent means ± SEM of three assays performed in triplicate. Values were normalized to controls in b and c. (*p<0.05; paired t-test).

**Figure 7:**
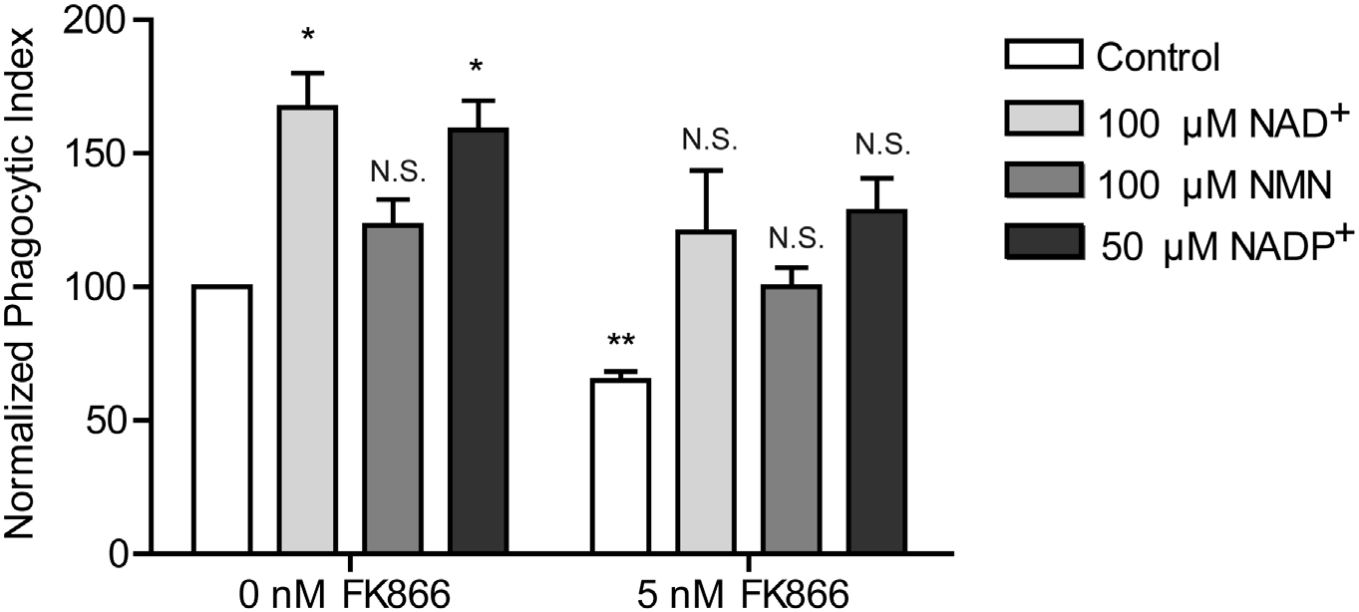
NAD^+^, NMN, and NADP^+^ supplementation stimulate phagocytosis and give reversal of FK866 effects. Cells were incubated for 24 hours with 100 µM NAD^+^, 100 µM NMN or 50 µM NADP^+^ in the presence or absence of 5 nM FK866 and activated overnight with LPS before they were incubated with FITC-labeled complement opsonized zymosan (COZ) particles for 30 min and analyzed by FACS. Values were normalized to the 0 nM FK866 control. Data represent normalized means ± SEM of four (0 nM FK866) or three (5 nM FK866) experiments performed in duplicate. (N.S., not significant, *p<0.05, **p<0.01; paired t-test).

Finally, we assessed NAD^+^’s role in phagocytosis, the most typical immune function of macrophages which requires special morphodynamic activity. The mechanism of phagocytosis shares many similarities with that of adhesive spreading of cells [57]. Intracellular uptake of FITC-labeled, complement (serum) opsonized zymosan (COZ particles) by RAW 264.7 macrophages was impaired after 15 and 24 hour FK866 treatment (Figure 6A–C). Both the percentage of cells that engaged in phagocytosis (Figure 6A) and the mean fluorescence intensity of active cells (Figure 6B) were significantly reduced at these two time points. Of note, at 3 hours of incubation with FK866 we observed no effect, although the NAD^+^ level was already significantly lowered (Figure 1B). In Maf-DKO cells, phagocytosis of COZ was assessed after 24 hour FK866 incubation and also found to be significantly inhibited (Figure S3), indicating that both these cell lines rely on NAMPT-mediated NAD^+^ synthesis for efficient phagocytosis. In addition, supplying cells with extracellular NAD^+^, NMN, or NADP^+^ stimulated phagocytosis of COZ and prevented this inhibitory effect of FK866 (Figure 7). Although it is not exactly clear how NAD^+^ and NADP^+^ are taken up by the cell, this result further supports our idea that NAD^+^ is a principal compound in the control of phagocytosis efficiency and confirms that FK866 exerts its effect on morphofunctional activity via a reduction in concentration of this pyridine nucleotide.

As the number of actively phagocytosing cells and the number of COZ particles engulfed per cell (Figure 6A and B, respectively) do not provide information about the internalization efficiency, i.e. the fraction of bound COZ particles that is actually engulfed, we decided to perform also an internalization efficiency assay. Since the inhibitory effects of 15 and 24 hour NAMPT inhibition were comparable, we treated cells with FK866 for the entire period of 24 hours. After incubation with COZ, the extracellular particles were not removed by lyticase, but samples were split and the fraction of internalized particles determined from the signal ratio between trypan blue quenched (internal) and total unquenched fluorescence (see Materials and Methods for details). Figure 6D shows that the ability to internalize particles was not compromised in the presence of FK866. The association with the phagocytic target is a crucial early step in phagocytosis and is mediated by multiple actin-rich membrane structures (filopodia, pseudopodia, and membrane ruffles) that are displayed on the cell surface [58]–[60]. Macrophages use these protrusive structures to probe the environment and capture targets for phagocytosis. When combined, the findings listed in Figures 4 and 5, together with the observation that fewer cells engaged in phagocytosis (Figure 6A) and their internalization ability was not affected (Figure 6D), suggest that macrophages treated with FK866 may have lost the ability to capture COZ efficiently, perhaps by loss of the ability to form proper membrane protrusions and adherence structures. Once contact between cell and particle is established, [NAD^+^] variation apparently has no effect on successful internalization.

## Discussion

**Figure 8:**
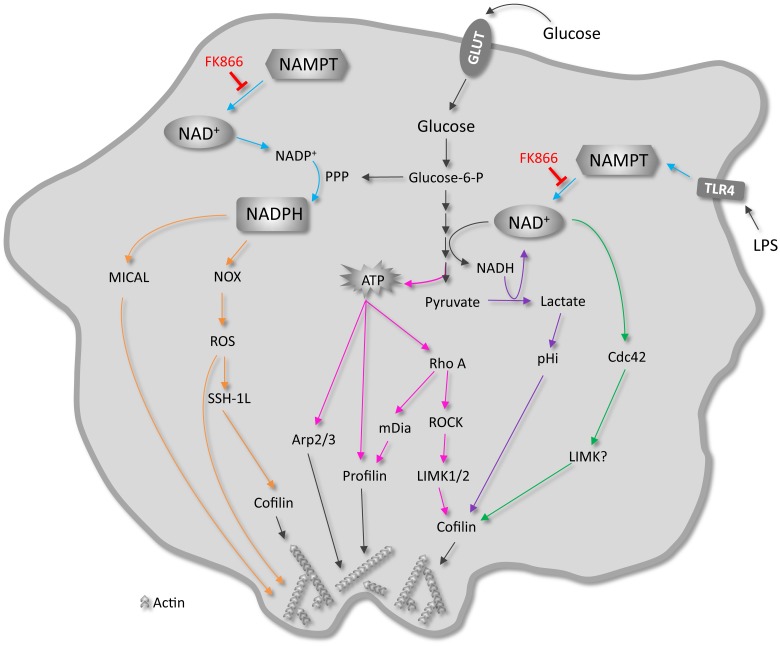
NAMPT-mediated salvage synthesis of NAD^+^ controls morphofunctional changes of macrophages: Synthesis of literature evidence. NAD^+^ is required to sustain high glycolytic activity in macrophages. Metabolism of glucose yields cellular ATP which is loaded onto G-actin monomers by profilin before incorporation into an actin filament, or used to activate the Arp2/3 complex. ATP also binds to Rho GTPase Rho A, which in turn also stimulates actin polymerization (pink arrows). LPS stimulation induces the expression of the NAD^+^ synthesizing enzyme NAMPT (turquoise arrows). NAMPT-mediated NAD^+^ synthesis may regulate actin dynamics by activating Cdc42 (green arrows) or by influencing cellular metabolism and, thereby, intracellular pH and cofilin activity (purple arrows). Alternatively, NAMPT may control redox regulation of the actin cytoskeleton via mical or NADPH oxidase by regulating NADPH levels (orange arrows). GLUT, glucose transporter; NOX, NADPH oxidase; PPP, pentose phosphate pathway; ROS, reactive oxygen species; SSH-1L, slingshot-1L; TLR4, toll-like receptor 4.

Macrophage function involves efficient migration into tissues, adhesion to the extracellular matrix, probing or sampling of their extracellular environment via filopodia, and – most importantly - phagocytosis of the pathogens and foreign particles they encounter. These processes rely on proper regulation of polymerization and depolymerization, branching and debranching of the actin cytoskeleton and on adequate cellular ATP supply, but the precise metabolic requirements and mechanisms involved are not yet fully understood. The NAD^+^ biosynthetic enzyme NAMPT has been implicated in linking NAD^+^ metabolism and the inflammatory cytokine response of monocytes and macrophages and to play a regulatory role in cancer cell motility [30]–[33]. Here we have investigated the importance of NAMPT dependent NAD^+^ synthesis for the actin driven processes of adhesion and spreading and phagocytosis in macrophages. As a novel finding we report that these morphofunctional activities depended heavily on NAD^+^ availability, even under metabolic conditions whereby cell growth and viability and intracellular ATP levels appeared not (yet!) affected. How these metabolic and morphodynamic activities are mechanistically controlled remains to be further investigated, but possibilities are discussed further below and graphically summarized in Figure 8.

Although the biosynthetic pathways involved in the synthesis of NAD(H) and NADP(H) are generally quite well conserved in mammalian cells, their synthetic capacity and also the intracellular levels of NAD(H) and NADP(H) may differ dramatically between cell types [21], [61]. NAD^+^ can be produced via *de novo* synthesis from tryptophan or via three salvage pathways. It also serves as precursor for the other three pyridine nucleotides, NADH, NADP^+^, and NADPH. Its reduced form, NADH, is produced in reactions catalyzed by dehydrogenases (mainly by LDH and in the TCA cycle) while NADP^+^ is synthesized through the phosphorylation of NAD^+^ by NAD kinase (NADK). NADP^+^ is subsequently and very quickly reduced, so that cellular NADP(H) occurs mostly as NADPH [62]. In contrast, cellular NAD(H) occurs mainly in the oxidized form, i.e. NAD^+^. Our measurements shown in Figure 1 confirm that this general picture also holds for RAW 264.7 and Maf-DKO macrophages. However, one striking observation was that RAW 264.7 cells contain remarkably high levels of NADP(H) and appear to maintain these levels during FK866 treatment. FK866 inhibition caused a marked reduction in NAD^+^ level already after 3 hours of treatment, while NADP(H) levels were not significantly affected. At later time points, NAD^+^ and NADH were totally depleted while 40% of the cellular NADPH still remained. That cellular NAD(H) is more sensitive to NAMPT inhibition by FK866 was also observed for the Maf-DKO cell line and for U251 glioma cells and activated T cells in previous studies [33], [63]. Although the mitochondrial NAD(P)(H)-pool was not left completely untouched, it was clearly less sensitive to NAMPT inhibition. More importantly, FK866-mediated NAMPT inhibition did not noticeably affect mitochondrial respiration. Multiple divergent explanations are possible for these combined observations. Firstly, mitochondria may not be solely dependent on NAMPT activity to build up a pool of NAD^+^. Some studies show that these organelles do not even contain NAMPT themselves, although this is an issue of some controversy [49], [64]. According to Nikiforov et al. [50], NMNAT3 is the only enzyme of NAD^+^ synthesis in mitochondria of human cells. This enzyme uses NMN, formed by cleavage of cytosolic NAD^+^, as substrate for intramitochondrial synthesis of NAD^+^. However, a more recent study by Felici et al. [51] contradicts these assertions showing that cytosolic NAD^+^ maintains the mitochondrial NAD^+^ pool and that neither NMNAT3v1 nor its splice variant FKSG76 is detectable in human cells. Moreover, upon of transfection of NMNAT3v1, FKSG76 was the isoform found to be localized to the mitochondria but to be involved in NAD^+^ splicing rather than synthesis. What remains, is that the mitochondrial NAD(P)(H) pool in our macrophage model cells reacts differently, but appears not to be completely separated from the cytosolic pool. Therefore, the reduction in mitochondrial autofluorescence signal (Figure 2D) can be best explained as an effect of a drop in cytosolic NAD^+^.

The global NAD^+^ depletion after 24 hours resulted in a reduced glycolytic rate but the cells nevertheless managed to maintain cellular ATP production. How this is accomplished is currently not completely clear to us. Recently Tan et al. [65], showed that FK866 treatment of cancer cells led to an attenuation of glycolysis at the GAPDH reaction resulting in a carbon overflow into the pentose phosphate pathway, a reduced carbon flow into the TCA cycle, and eventually a reduction in cellular ATP. They also showed that the inhibitory effect on the TCA cycle was less when glutamine was not limiting in the culture medium. Since our cells were cultured in the presence of glutamine and glycolysis was clearly inhibited, glutamine consumption may have been upregulated in the presence of FK866. Upon glutaminolysis, glutamine is converted into pyruvate and, finally, lactate via the formation of TCA-cycle intermediates α-ketogluterate, succinate, fumarate, and malate, yielding NADH and FADH_2_ for ATP production via OXPHOS. This could also explain the minor increase in lactate formation from 1.5 to 1.8 moles of lactate per mole of glucose consumed (ratio of bars, Figure 2A, B). We did not observe any increase in basal mitochondrial OXPHOS activity (Figure 2E), however, indicating that ATP production was not significantly upregulated via this route. Thus, any increase in glutaminolysis probably only matched the reduced carbon flow from glycolysis into the TCA cycle but did not contribute to ATP homeostasis.

Can we thus rule out limited availability of ATP, due to lack of NAD^+^, as the cause of morphofunctional change in FK866 treated macrophages? Earlier we showed that microcompartmentation of ATP-supply plays a role in the control of cell migratory activity of cells [66]. It remains therefore possible that FK866-mediated NAD^+^ reduction is associated with a local change in ATP supply capacity, which may be not sufficient to meet the highly variable and transient demand for cytoskeletal remodeling and phagocytosis. Under these conditions global cellular ATP concentrations may not be overtly affected. Interestingly, after 3 hours of NAMPT inhibition still no inhibitory effect was observed on either phagocytosis, spreading, or LPS induced morphology change, while global NAD^+^ was already significantly reduced. In addition, the accumulated glucose consumption and lactate production over the entire 24 hour incubation period was not significantly different for control and FK866 treated cells (results not shown). The decrease in glycolytic flux only became substantial towards the end of the 24 hour treatment (Figure 2A, B). Thus, if metabolic coupling of NAD^+^ concentration to local or global ATP supply is not the primary mechanism that accounts for FK866 effects on macrophage morphodynamics, what other mechanisms could be involved?

Apart from limiting the ATP supply, NAD^+^-depletion may have other metabolic consequences that could (directly or indirectly) interfere with actin dependent morphodynamics. For example, the production of lactate in the last step of glycolysis, together with release of protons from the conversion of NADH to NAD^+^, influences global intracellular pH (pHi). Locally, the lactate molecules, when transported out of the cell by the monocarboxylate transporter (MCT1) together with the H^+^ [67], [68], form a pH gradient across the membrane, which is thought to induce cytoskeletal rearrangement via actin-binding proteins gelsolin, cofilin, and talin [69]. By inhibiting the glycolytic flux to lactate, NAD^+^-depletion may thus exert direct effects on global and local pH homeostasis. In line with this, we have recently shown that both the pHi and the pericellular pH (pHe) of FK866 treated and NAMPT knockdown U251 cells were elevated compared to control cells and that the addition of lactic acid to culture media restored the inhibitory effect of FK866 on U251-shNAMPT cell migration [33]. Whether [NAD^+^] changes also cause shifts in lactate dehydrogenase (LDH) activity in macrophages, and thereby affect the remodeling of the acto-myosin cytoskeleton in the micro-environment of phagocytic cup formation remains a topic for further study.

Upon stimulation with LPS, macrophages undergo dynamic morphological changes and reorganization of their actin cytoskeleton [8], [9]. LPS forms a complex with LPS binding protein (LBP) present in serum, binds to CD14, and finally to the TLR4/MD-2 complex on the macrophage cell membrane [70], [71]. LPS signaling via TLR4 induces Src family kinase mediated phosphorylation of Pyk2 and paxillin [8], [9], [72] as well as phosphorylation of the PI3K/AKT pathway [73]. These are molecules acting upstream of the small GTPases Rho, Rac and Cdc42 which regulate stress fiber, lamellipodia and membrane ruffle, and filopodia formation, respectively [74]–[76]. As a consequence, LPS stimulation induces cell spreading and the formation of protrusive membrane structures. In addition, LPS is responsible for the activation of the complement receptor (CR3) that is involved in the recognition of serum opsonized particles via Rap1 and RhoA [77], [78]. Together, this enhances the phagocytic capacity of macrophages. We observed here that NAD^+^-depletion upon NAMPT inhibition markedly reduced the phagocytic capacity of RAW 264.7 and Maf-DKO cells, even though they were still able to internalize particles once these were bound. Recently, NAMPT was shown to regulate directionally persistent migration of vascular smooth muscle cells (SMCs), mediated by Cdc42 activation [79]. Although Cdc42 has been implicated in IgG-mediated and not CR3-mediated phagocytosis [80], it is also involved in filopodia formation, the assembly of multimolecular focal complexes [76], and in integrin-mediated adhesion and spreading [81] by regulating actin network reorganization via LIMK2-mediated phosphorylation/inactivation of cofilin [82]. This, in combination with the knowledge that phagocytosis is an area-restricted form of cell adhesion and spreading [57], suggests that our findings can also be explained by direct NAMPT activity effects on the Cdc42-mediated probing, particle capturing, and adhesion activity of macrophages.

Finally, yet another determinant of proper actin cytoskeletal remodeling is cellular redox state [53], [54], in which NAD^+^/NADH and NADP^+^/NADPH play an important role. In macrophages, cellular NADPH may influence redox regulation of actin remodeling in several ways. Professional phagocytes express NADPH oxidases (NOX) which use NADPH to transfer an electron to molecular oxygen during the respiratory burst. This gives rise to superoxide which can further be converted into hydrogen peroxide (H_2_O_2_) and other reactive oxygen species (ROS), both intra- and extracellularly. Extracellularly and in the phagosomal lumen, these ROS serve a microbicidal function, thereby offering protection against pathogens. Intracellularly, the ROS is involved in redox signaling, mediating the production of cytokines via NFκβ-dependent mechanisms [83] but also regulating the activity of cofilin [84] which increases the recycling of actin monomers at the pointed end of actin filaments. At the same time, NADPH is also responsible for the regeneration of antioxidant molecules such as glutathione (GSH) and thioredoxin (Trx), thereby offering protection against oxidative stress, which may affect the redox state of actin itself [85]. NADPH may also regulate the balance between ROS-mediated activation/deactivation of signaling molecules (GTPases) that control the actin cytoskeleton [54]. Furthermore, NADPH is required for the disassembly of actin filaments by MICAL redox enzymes [86] which have been shown to regulate actin dynamics in many cell types [87]–[90], including macrophages [91]. Our findings suggest that NADP(H) may even be a principal pyridine nucleotide in coupling NAD^+^-metabolism to actin-based morphodynamics, based on the following arguments: i) RAW 264.7 cells have a relatively high level of cellular NADPH, ii) NADP(H) levels were relatively spared during the initial phases of FK866 inhibition and - in association - morpho-functional changes became only apparent at later time points when cellular NADPH reached critical levels, i.e. after 6, 15, or 24 hours of NAMPT inhibition, and finally, iii) supplementation with NADP^+^ quite effectively rescued phagocytosis in FK866 treated cells (Figure 7).

Studies during the last decade have identified FK866 as a promising drug against many different cancers. Currently, this compound is being tested in phase two clinical trials [92]–[94]. Tumor cells are more sensitive to inhibition of NAD^+^ synthesis than normal cells since they are metabolically highly glycolytic and, therefore, FK866 is an effective inhibitor of tumor cell growth. Earlier we have shown in glioma cells [33], and here now also in macrophages, that morphodynamic behavior (motility, phagocytosis, spreading) is significantly inhibited at moderately effective FK866 concentrations, during treatment periods wherein proliferation and viability were not yet affected. When extrapolating these findings to the *in vivo* situation, we expect that the magnitude of effects of FK866 will be dependent on a tumor-cell’s nutrient state and its capacity for NAD^+^ synthesis via different metabolic pathways (i.e. via *de novo* or salvage pathways, via NAPRT or NAMPT). Treatment with FK866 may thus have broad consequences, particularly also for normal glycolytic cells in tumors such as macrophages. For example, our findings thus leave open the possibility that FK866 could affect the morphodynamics of tumor associated macrophages (TAMs), which have a promoting role in tumor cell migration [95], tumor cell proliferation and angiogenesis [96]. From a treatment perspective, this could help to further enhance FK866’s anti-cancer effect. On the other hand, FK866 treatment could have adverse effects and lead to an overall repression of macrophage migratory and phagocytic function, with less clearance of apoptotic cells and debris from dying tumors. These effects – even in a more broader sense – are also a point of concern for use of FK866 as anti-inflammatory drug for the treatment of autoimmune diseases such as rheumatoid arthritis, inflammatory bowel disease, or Crohn’s disease. Further study in patients and animal models *in vivo* is, therefore, necessary to fully understand all possible consequences of FK866 treatment regimes.

In summary, we report here that FK866 mediated inhibition of NAMPT causes NAD^+^-depletion and down regulation of glycolysis in macrophage model cells. Inhibition of NAD^+^ salvage synthesis did not affect cell viability, proliferative capacity, or ATP levels but significantly impaired morphofunctional changes involved in phagocytosis and spreading. Future research should elucidate how NAD^+^ production and these processes are exactly linked.

## Supporting Information

Figure S1 **RAW 264.7 and Maf-DKO proliferation.** A, Proliferation of RAW 264.7 and Maf-DKO cells in the presence and absence of L929 cell conditioned medium (M-CSF). B, RAW 264.7 proliferation in the presence of FK866 over a period for 72 hours. Proliferation was monitored by measuring the increase in protein mass at the indicated time periods. Data represent means of three independent experiments performed in triplicate. (*p<0.05, **p<0.01; unpaired t-test).
(TIF)

Figure S2 **RAW 264.7 and Maf-DKO morphology and actin structures are affected during NAD^+^-depletion.** RAW 264.7 (A–D) and Maf-DKO (E–H) macrophages were seeded on glass coverslips in RAW264.7 (without M-CSF) and Maf-DKO (with M-CSF) medium, respectively. Cells were incubated for 24 h in control 5 nM FK866 medium and stimulated o/n with 100 ng/ml LPS or left unstimulated. Cells were fixed in 2% PFA, stained with phalloidin-Alexa568, and imaged on a Zeiss LSM510 META confocal laser scanning microscope.
(TIF)

Figure S3 **FK866-mediated NAD^+^-depletion reduces RAW 264.7 and Maf-DKO phagocytosis efficiency.** Cells were seeded in medium containing 20% conditioned medium from L929-cell cultures (+M-CSF) and incubated for 24 hours in the presence or absence of 5 nM FK866. Cells were additionally stimulated overnight with 100 ng/ml LPS. After 30 minutes incubation with FITC-labelled complement opsonized zymosan (COZ) particles, cells were harvested, fixed, and analyzed by FACS. The percentage of FITC positive cells were measured as well as the mean fluorescence of this population. The product of these two parameters were used to calculated the phagocytic index. Data represent normlaized means ± SEM of three experiments performed in duplicate. (*p<0.05; paired t-test).
(TIF)
