# Supplementary figures and images for: NAMPT-Mediated Salvage Synthesis of NAD+ Controls Morphofunctional Changes of Macrophages
Source: PLoS One. 2014 May 13;9(5):e97378. doi: 10.1371/journal.pone.0097378 (PMC4019579; doi:10.1371/journal.pone.0097378)

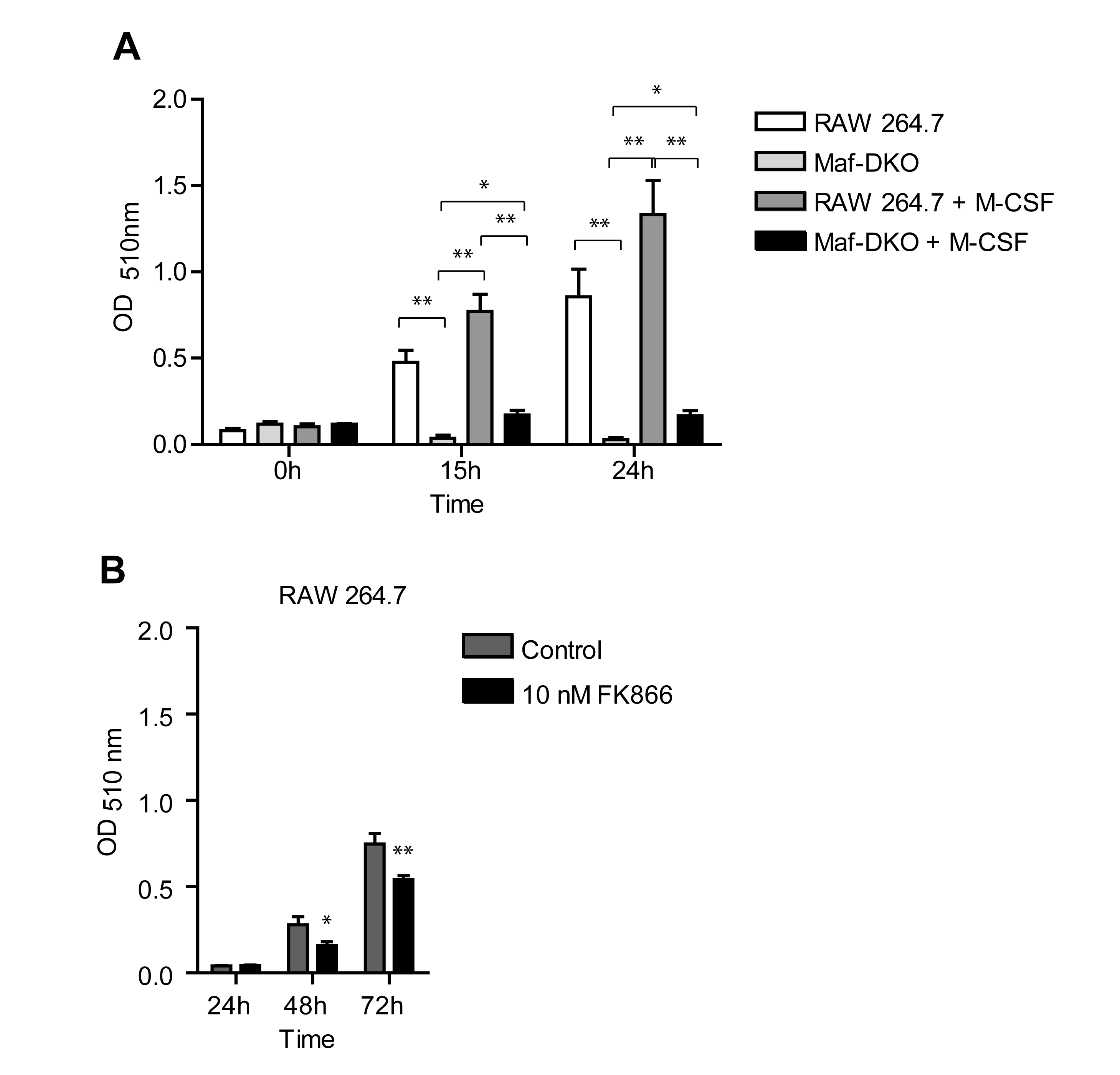

Supplement: Figure S1 — RAW 264.7 and Maf-DKO proliferation. A, Proliferation of RAW 264.7 and Maf-DKO cells in the presence and absence of L929 cell conditioned medium (M-CSF). B, RAW 264.7 proliferation in the presence of FK866 over a period for 72 hours. Proliferation was monitored by measuring the increase in protein mass at the indicated time periods. Data represent means of three independent experiments performed in triplicate. (*p<0.05, **p<0.01; unpaired t-test). (TIF) [file pone.0097378.s001.tif]

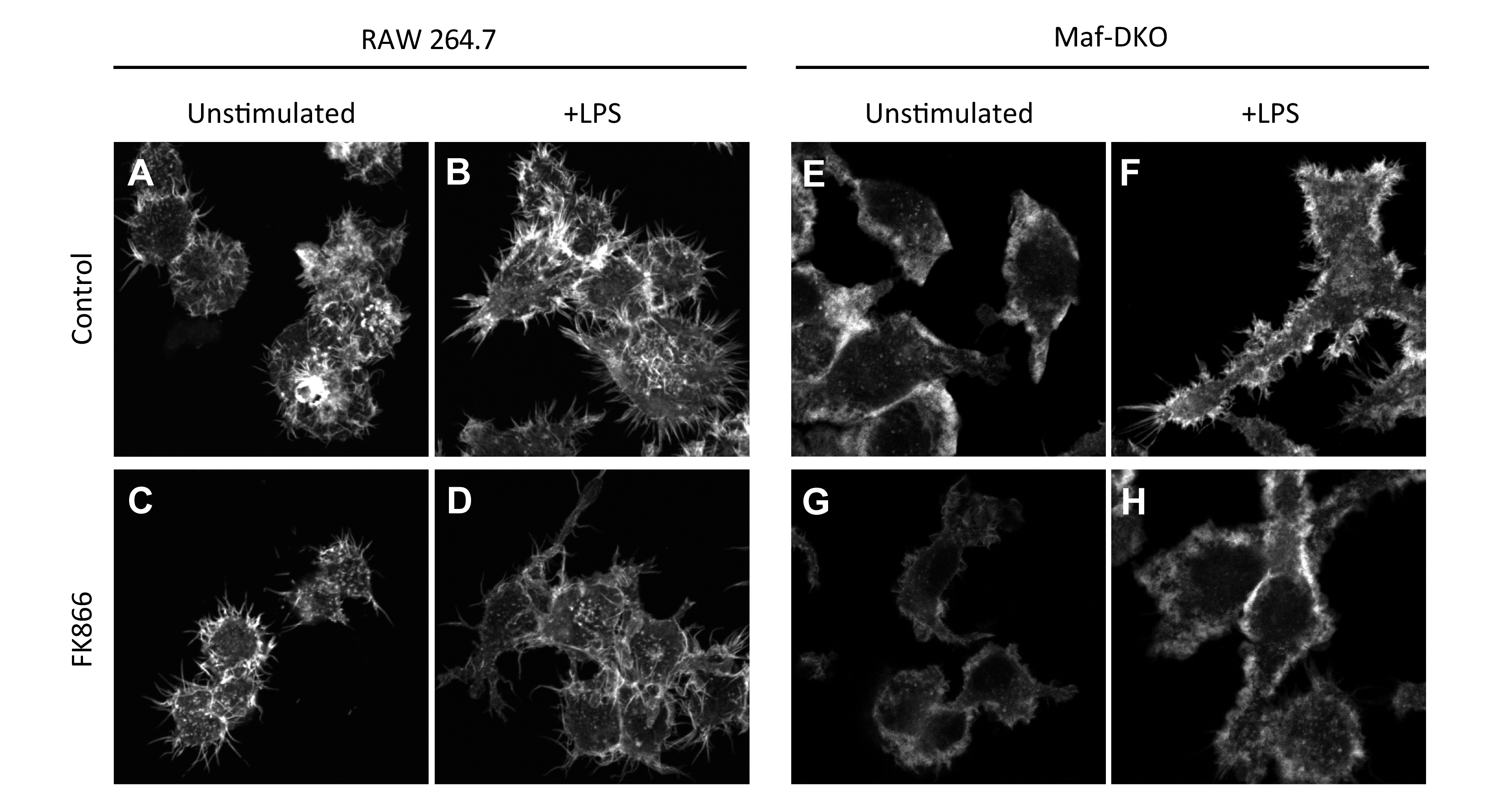

Supplement: Figure S2 — RAW 264.7 and Maf-DKO morphology and actin structures are affected during NAD+-depletion. RAW 264.7 (A–D) and Maf-DKO (E–H) macrophages were seeded on glass coverslips in RAW264.7 (without M-CSF) and Maf-DKO (with M-CSF) medium, respectively. Cells were incubated for 24 h in control 5 nM FK866 medium and stimulated o/n with 100 ng/ml LPS or left unstimulated. Cells were fixed in 2% PFA, stained with phalloidin-Alexa568, and imaged on a Zeiss LSM510 META confocal laser scanning microscope. (TIF) [file pone.0097378.s002.tif]

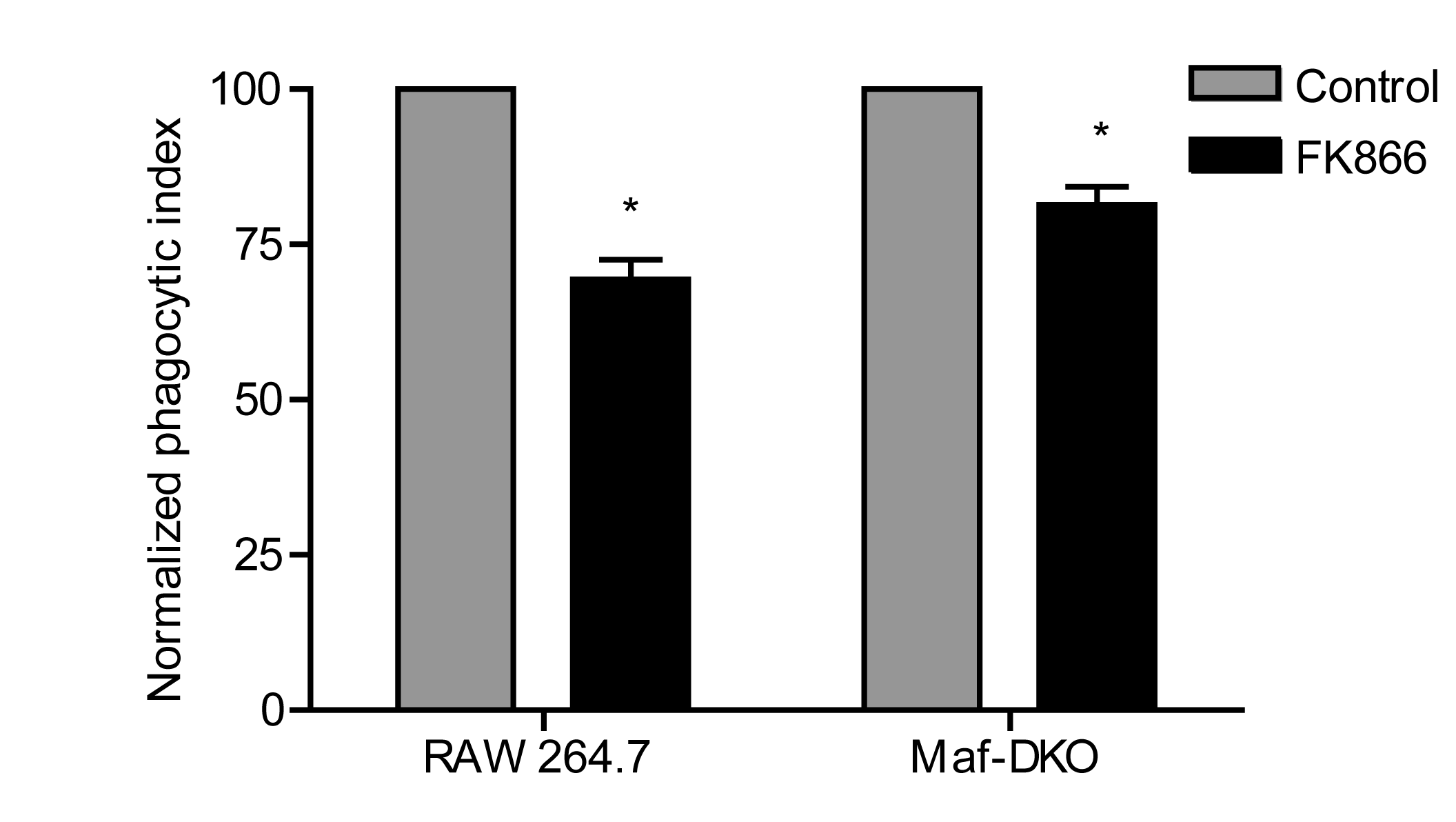

Supplement: Figure S3 — FK866-mediated NAD+-depletion reduces RAW 264.7 and Maf-DKO phagocytosis efficiency. Cells were seeded in medium containing 20% conditioned medium from L929-cell cultures (+M-CSF) and incubated for 24 hours in the presence or absence of 5 nM FK866. Cells were additionally stimulated overnight with 100 ng/ml LPS. After 30 minutes incubation with FITC-labelled complement opsonized zymosan (COZ) particles, cells were harvested, fixed, and analyzed by FACS. The percentage of FITC positive cells were measured as well as the mean fluorescence of this population. The product of these two parameters were used to calculated the phagocytic index. Data represent normlaized means ± SEM of three experiments performed in duplicate. (*p<0.05; paired t-test). (TIF) [file pone.0097378.s003.tif]
